# Estimating coverage of a women’s group intervention among a population of pregnant women in rural Bangladesh

**DOI:** 10.1186/1471-2393-12-60

**Published:** 2012-06-29

**Authors:** Layla Younes, Tanja AJ Houweling, Kishwar Azad, Anthony Costello, Edward Fottrell

**Affiliations:** 1 UCL for International Health and Development, Institute of Child Health, 30 Guildford Street, London, WC1N 1EH, UK; 2 Perinatal Care Project, Diabetic Association of Bangladesh, 122 Kazi Nazrul Islam Avenue, Dhaka, 1000, Bangladesh; 3 Dept. of Public Health, ErasmusMC University Medical Center Rotterdam, P.O. Box 2040, 3000 CA, Rotterdam, The Netherlands

## Abstract

**Background:**

Reducing maternal and child mortality requires focused attention on better access, utilisation and coverage of good quality health services and interventions aimed at improving maternal and newborn health among target populations, in particular, pregnant women. Intervention coverage in resource and data poor settings is rarely documented. This paper describes four different methods, and their underlying assumptions, to estimate coverage of a community mobilisation women’s group intervention for maternal and newborn health among a population of pregnant women in rural Bangladesh.

**Methods:**

Primary and secondary data sources were used to estimate the intervention’s coverage among pregnant women. Four methods were used: (1) direct measurement of a proxy indicator using intervention survey data; (2) direct measurement among intervention participants and modelled extrapolation based on routine longitudinal surveillance of births; (3) direct measurement among participants and modelled extrapolation based on cross-sectional measurements and national data; and (4) direct measurement among participants and modelled extrapolation based on published national data.

**Results:**

The estimated women’s group intervention’s coverage among pregnant women ranged from 30% to 34%, depending on method used. Differences likely reflect differing assumptions and methodological biases of the various methods.

**Conclusion:**

In the absence of complete and timely population data, choice of coverage estimation method must be based on the strengths and limitations of available methods, capacity and resources for measurement and the ultimate end user needs. Each of the methods presented and discussed here is likely to provide a useful understanding of intervention coverage at a single point in time and Methods 1 and 2 may also provide more reliable estimates of coverage trends.

**Footnotes:**

^1^Unpublished data from three focus group discussions with women’s group members and facilitators participating in the Women’s Groups intervention.

## Background

Progress towards Millennium Development Goals four and five of reducing child and maternal morality requires focused attention on better access, coverage and utilisation of good quality maternal and reproductive health services and interventions [1-3]. Understanding coverage of population health interventions and services is important in monitoring their implementation and receipt and assessing whether they reach the intended target population(s). Understanding coverage also enables the assessment of whether certain target groups are being reached more effectively than others. Coverage levels and trends are, therefore, helpful to assess the performance of health systems, services and interventions. They guide policy makers in planning strategies to improve health by identifying gaps in health systems and resources that require focused attention [4-6].

Coverage indicators are quantitative in nature and typically express the proportion of a target population exposed to or receiving a health intervention. In their most simple form, coverage indicators are calculated by dividing the number of individuals receiving an intervention by the total population eligible for that intervention [7]. For example, coverage indicators of maternity care include the proportion of pregnant women who received at least two antenatal care visits, the proportion of deliveries occurring in a health facility, the proportion of deliveries with skilled attendance at birth, and the proportion of women attended at least once during the postpartum period by skilled health personnel [7]. Clear definitions and measurements of the numerator and the denominator for these proportions are therefore crucial.

Methods to estimate intervention coverage in resource- and data-poor settings are rarely described or documented and measuring coverage among pregnant women can be particularly challenging. In settings with weak or non-existent vital registration systems and where records of population numbers, uptake of services or exposure to interventions are scarce, estimating intervention coverage demands creative use of primary and secondary data sources. This paper describes four different methods, and their underlying assumptions, to estimate population coverage of a women’s group community mobilisation intervention for maternal and newborn health in rural Bangladesh. The advantages and disadvantages of each method are discussed.

## Methods

The women’s group community mobilisation intervention is delivered in three districts in Bangladesh and is described in detail elsewhere [8]. Essentially, the intervention is comprised of monthly women’s group meetings whereby communities prioritise problems that threaten the health of mothers and their babies and plan, implement and evaluate strategies to overcome these problems. Local women, who were trained in participatory methods and provided with basic information about maternal and newborn health, facilitate a participatory learning and action cycle.

The target population for the intervention is pregnant women and so all ever-married women of reproductive age are invited to participate. Every woman’s group meeting, however, is open to participation by anybody in the community, including men. Open participation is crucial to the community’s acceptance of the women’s group intervention and the dissemination of key health facts. Specific strategies were employed to attract women of reproductive age, pregnant or newly married women to participate in the groups. These strategies included actively approaching pregnant and newly married women in the community, providing them with information about the nature and objectives of the women's group intervention and encouraging them to participate.

A prospective surveillance system is in place to record and monitor birth outcomes in intervention areas. Traditional Birth Attendants (TBAs) act as key informants to notify a project monitoring and evaluation (M&E) team of all births and deaths to women of reproductive age in the intervention areas. Monitors verify births and deaths, and all women who gave birth are approached to be interviewed. Structured interviews gather detailed data on birth outcomes and care before, during and after delivery and are conducted between 42 days and one year after delivery using a survey. Detailed quantitative and qualitative process evaluation information on intervention processes, context and participation, particularly of pregnant women, is also collected throughout the intervention period. The third source of primary data gathered by the intervention implementation team is an annual household census, which records the total number of women of reproductive age and children living in the intervention areas. Publicly available secondary population data sources in Bangladesh include the 2001 Bangladesh Maternal Health Services and Maternal Mortality Survey (BMMS) [9], the 2007 Bangladesh Demographic and Health Survey (BDHS) [10] and the Bangladesh Bureau of Statistics (BBS) national census data from 2001 [11].

Four methods have been developed to estimate intervention coverage among pregnant women, each using at least one of the primary and/or secondary data sources mentioned above. Confidence intervals for all estimates are calculated using the confidence interval calculator in STATA version 12. Coverage estimates that are based on secondary data and assumptions about underlying fertility levels are presented with ranges that reflect extreme values and uncertainty in these assumptions. Estimates derived from each method are compared and critically discussed in terms of these inherent assumptions, methodological strengths and weaknesses and differing data and resource demands. All estimates are for the period of October 2009 to May 2010.

### Method 1: Direct measurement of a proxy indicator

Recognising that direct measurement of the pregnancy status of all women in the study area is unfeasible, the proportion of all deliveries in which the mother participated in the women’s group intervention can be measured using survey methods and subsequently used as a proxy-measure for coverage among pregnant women. Method 1 uses data from the community monitoring and evaluation (M&E) survey whereby all deliveries in the study areas are recorded and all women are invited to participate in a survey interview. Women’s participation in the women’s group intervention is ascertained through these interviews. The proportion of women reporting participation during pregnancy approximates the exposure among pregnant women in the population and can be presented with 95% confidence intervals.

### Method 2: Direct measurement among participants and modelled extrapolation based on routine surveillance of births

During each monthly woman’s group meeting, group facilitators ask women to volunteer their pregnancy status. This count provides the numerator in the calculation of coverage among pregnant women at a particular point in time. Obtaining the same information for the denominator, i.e. the total number of currently pregnant women in the whole population is more complex and arguably not feasible within a short time frame and without considerable resources or ethical implications. However, assuming that fertility levels remain fairly constant in relatively small geographic areas over a nine-month period, one can assume that the total number of babies born is approximately equal to the total number of pregnancies conceived and, therefore, that for every pregnancy that ends, there are nine others that are in gestation. The count of all births occurring in the study area from the prospective birth surveillance can therefore be considered an approximation of the number of new pregnancies arising, which, if multiplied by nine (for the average duration of pregnancy in months) gives an approximation of the total number of pregnant women in the population. In theory, therefore, the minimum intervention coverage among pregnant women can be estimated by dividing the number of pregnant women participating in women’s groups by the total number of deliveries that month multiplied by nine.

In practice, however, few women are aware of their pregnancy status from the moment of conception and fewer are willing to share their pregnancy status during the early stages of pregnancy. This is certainly the case in rural Bangladesh where cultural norms and beliefs usually prevent women from publicly disclosing their pregnancy status until after approximately 3.5 months. Rather than multiplying the denominator by nine, therefore, it is arguably more sensible to multiply by 5.5 (i.e. 9 minus 3.5). So, to estimate intervention coverage among pregnant women, the monthly count of currently pregnant women participating in women’s groups is divided by the total number of births captured multiplied by 5.5.

To test the sensitivity of this measure to varying assumptions of the duration of pregnancy concealment, plausible minimum coverage can be estimated by multiplying the denominator by 8 to represent early disclosure after just one month of pregnancy whilst maximum coverage may be estimated by multiplying the denominator by 4, representing delayed pregnancy status disclosure until pregnancy may be noticeable by others at approximately 5 months. These extremes therefore represent a range of coverage estimates depending on underlying assumptions of duration of pregnancy concealment, each of which can be presented with 95% confidence intervals.

### Method 3: Direct measurement among participants and modelled extrapolation based on cross-sectional measurements and national data

As in Method 2, monthly counts of currently pregnant women participating in the women’s group intervention provides the numerator in calculating coverage among pregnant women at a particular point in time. However, rather than basing the denominator on counts of births, which demands prospective community surveillance or birth registration systems, Method 3 uses the intervention’s household census data and national BDHS data to estimate the total number of pregnant women in the study area. An annual household census, implemented by the study team, records the total number of households and their inhabitants, by age and sex, residing in the study area. To estimate the denominator (i.e. the total number of currently pregnant women in the study area), the total number of married women in reproductive age identified through the intervention’s household census is multiplied by the 2007 BDHS estimate of the proportion of women of reproductive-age who reported being pregnant at the time of the survey, which is 5.6% in rural areas [10]. This method assumes that overall fertility rates have not changed substantially since 2007. However, fertility rates do vary by administrative divisions and the proportion of currently pregnant women ranges from 4.9% in Dhaka and Khulna divisions to 6.9% in Sylhet division. This range in the proportion of currently pregnant women can be used to calculate plausible minimum and maximum coverage by deriving the denominator from the lower and upper estimates of proportions of pregnant women, respectively, each of which can be presented with 95% confidence intervals.

### Method 4: Direct measurement among participants and modelled extrapolation based on published national data

Often, public health interventions may not have the resources or capacity to collect and process data. Nevertheless, available data that have been previously published on a regional or national level can be used to provide estimates of intervention coverage. The count of currently pregnant women participating in the women’s group intervention forms the numerator in calculating coverage among pregnant women at a particular point in time. The data capture demands for this are minimal and it is realistic to expect most services or interventions to keep a record of the number of recipients. The denominator is derived from the total number of married women in reproductive age as identified through the 2001 BBS national census multiplied by the 2001 Bangladesh Maternal Health Services and Maternal Mortality Survey estimate of the proportion of women of reproductive-age who reported being pregnant in the same year (2001), which is 5.5 in rural areas [9]. As in method 3, the proportion of currently pregnant women varies across administrative divisions. Khulna division had the lowest proportion currently pregnant in 2001 (4.6%), whereas the highest proportion was reported for Sylhet division (6.4%). A plausible minimum coverage can therefore be estimated by multiplying the denominator by 6.4 whilst maximum coverage may be estimated by multiplying the denominator by 5 and 95% confidence intervals can be calculated for each estimate.

### Ethical Approval

The women’s group intervention trial and all monitoring and evaluation activities have received ethical approval from the University College London Research Ethics Committee (ID Number: 1488/001) and by the Ethical Review Committee of the Diabetic Association of Bangladesh. Informed verbal consent is obtained from all survey respondents before any data are collected.

## Results

A total of 45,820 eligible women (i.e. aged 15–49 years) reside in intervention areas and were indentified using the study’s household census. A total of 4046 women gave birth in the intervention areas between October 2009 and May 2010. Of the deliveries identified, a total of 3972 mothers were interviewed using the study’s M&E survey, a response rate of 98%. On average, 847 pregnant women were reported to have attended women’s group meetings each month from October 2009 to May 2010.

Coverage estimates varied from around 30% to 34% depending on method used. Table 1 shows the estimates of intervention coverage among pregnant women derived from each of the methods described above. For Method 2, upper and lower estimates based on variations in underlying assumptions in the calculation of the denominator caused estimates to range from as low as 21% to 42%, highlighting the sensitivity of this method to variations in the underlying assumption of duration of pregnancy concealment. Methods 3 and 4 were less sensitive to variations in the underlying assumptions.

**Table 1:** Estimates of Intervention’s Coverage Among Pregnant Women

**Method**	**Description**	**Numerator**	**Denominator**	**Estimate**
				**(95% CI)**
**1**	**Direct measurement of proxy indicator**	Deliveries to women who report participation in the women’s group intervention	Total number of deliveries where an interview was carried out	31%
				(29% -32%)
**2**	**Direct measurement among participants and modelled extrapolation based on routine surveillance of births**	Average number of participants in the women’s group intervention who reported being pregnant each month from Oct 2009 to May 2010	Average number of deliveries per month from Oct 2009 to May 2010 multiplied by 5.5	30%
				(30%-31%)
	· Minimum coverage	Average number of participants in the women’s group intervention who reported being pregnant each month from Oct 2009 to May 2010	Average number of deliveries per month from Oct 2009 to May 2010 multiplied by 8 (i.e. assuming a pregnancy concealment time of one month)	21%
				(21%-24%)
	· Maximum coverage	Average number of participants in the women’s group intervention who reported being pregnant each month from Oct 2009 to May 2010	Average number of deliveries per month from Oct 2009 to May 2010 multiplied by 4 (i.e. assuming a pregnancy concealment time of 5 months)	42%
				(41%-43%)
**3**	**Direct measurement among participants and modelled extrapolation based on cross-sectional measurements and nationaldata**	Average number of participants in the women’s group intervention who reported being pregnant each month from Oct 2009 to May 2010	Local household census-based number of women of reproductive-age (WRA) multiplied by BDHS estimate of the proportion of WRA who are currently pregnant, which is 5.6% in rural areas.	33%
				(31%-35%)
	· Maximum coverage	Average number of participants in the women’s group intervention who reported being pregnant each month from Oct 2009 to May 2010	Local household census-based number of women of reproductive-age (WRA) multiplied by the lower BDHS estimate of the proportion of WRA who are currently pregnant, which is 4.9% (Khulna and Dhaka divisions)	38%
				(36%-40%)
	· Minimum coverage	Average number of participants in the women’s group intervention who reported being pregnant each month from Oct 2009 to May 2010	Local household census-based number of women of reproductive-age (WRA) multiplied by the lower BDHS estimate of the proportion of WRA who are currently pregnant, which is 6.9% (Sylhet division)	27%
				(25%-28%)
**4**	**Direct measurement among participants and modelled extrapolation based national data**	Average number of participants in the women’s group intervention who reported being pregnant each month from Oct 2009 to May 2010	2001 National census reported number of women of reproductive-age multiplied by the 2001 BMMS estimate of the proportion of WRA who are currently pregnant, which is 5.5% (rural areas).	34%
				(33%-36%)
	· Maximum coverage	Average number of participants in the women’s group intervention who reported being pregnant each month from Oct 2009 to May 2010	2001 National census reported number of women of reproductive-age multiplied by the lower 2001 BMMS estimate of the proportion of WRA who are currently pregnant, which is 4.6% (Khulna division).	41%
				(39%-43%)
	· Minimum coverage	Average number of participants in the women’s group intervention who reported being pregnant each month from Oct 2009 to May 2010	2001 National census reported number of women of reproductive-age multiplied by the upper 2001 BMMS estimate of the proportion of WRA who are currently pregnant, which is 6.4% (Sylhet division).	30%
				(28% -31%)

Notes:

Total number of women in reproductive age: National Census 2001:44,662. Intervention’s Household Census 2009: 45,820.

Percentage currently pregnant in rural areas: BMMS 2001: 5.5 and BDHS 2007: 5.6.

## Discussion

Applying four different methods to estimate the coverage of a women’s group community mobilisation intervention among pregnant women in rural Bangladesh, coverage estimates ranged from 30% to 34%, depending on method used. Each of the methods presented has varying data and resource demands and is associated with specific strengths and limitations, which must be considered when selecting the most appropriate methods for end-user needs.

Method 1 depends on women accurately reporting participation in the women’s group intervention, and this may be subject to reporting bias during data capture. The method also makes the assumption that all pregnancies end in a delivery that is noticed and recorded by TBAs. This is unlikely to be true as early terminations through miscarriage or abortion are likely to be missed. The implication of this is an underestimation of the total number of pregnant women in the population and thus an overestimation of programme coverage. The survey methods used to gather data for Method 1 are also particularly resource demanding and require a reasonably high degree of competency from a monitoring and evaluation team for effective and efficient data processing. Such resources and competencies are unlikely to be available in many resource-poor settings. Nevertheless, in settings where resources and competency are available for direct measurement and provided that limitations of survey methods (such as recall and reporting bias) are recognised and minimised, direct measurement of proxy measures may be more informative and reliable than entirely modelled estimates.

Method 2 is largely based on the assumption that fertility remains constant in the intervention areas and study period and therefore the number of deliveries will equal the number of conceptions. Notwithstanding seasonal variations and longer-term fertility declines, this assumption may be reasonable given that the total fertility rate has not changed substantially on a year to year basis. The total fertility rate remained fairly stable at around 3.3 children per woman for most of the 1990s but declined to 2.7 according to the 2007 BDHS report [10].

As previously described, the numerator in Method 2 may be an underestimate of the true number of pregnant women participating in the women’s group intervention because some women who are in the early stages of pregnancy may not be aware of their pregnancy or may not want to disclose their pregnancy status. However, this is dealt with to a certain extent by adjusting the denominator. Furthermore, the fact that the denominator is likely to exclude pregnancies that end early due to miscarriages or abortions is also likely to minimise the problems of non-disclosure in the numerator since early termination of pregnancies and non-disclosure are both more likely to occur in the first trimester of pregnancy. Nevertheless, the upper and lower estimates generated by Method 2 indicate sensitivity to the assumed time before disclosure of pregnancy status and so formative work to inform this may be needed. Overall, Method 2 is appealing in that it is based on a prospective births surveillance system, thus enabling real-time, prospective monitoring of intervention coverage. However, such data processes and key informant systems are not trivial in terms of financial resources and technical capacity.

Methods 3 and 4 are progressively less resource- and data-demanding, using secondary data sources to estimate the number of pregnant women in the intervention areas. Although the numerator does require a degree of primary data capture, namely a register of women participating in the intervention and their pregnancy status, the technical demands for such data capture and processing are minimal and it is reasonable to expect that any intervention or service will keep a record of uptake. Both Methods 3 and 4 use secondary, cross-sectional data to derive the coverage estimate denominator. The cross-sectional nature of these data may be considered a limitation of these methods in that derived coverage estimates provide only a snapshot view and any changes in population size or fertility since the denominator data were captured cannot be taken into consideration. This is likely to affect the precision of any estimates. Furthermore, the application of national figures at a single point in time to relatively small geographical areas may mask regional variability and temporal fluctuations in population and fertility.

Unlike Method 2, it is not so simple to allow for non-disclosure of early pregnancy in coverage estimates in Methods 3 and 4 and thus the estimates derived from these methods may mis-represent programme coverage. Nonetheless, compromises of precision for low data and resource demands are likely to be acceptable to health programme and intervention managers wishing to approximate the coverage of their intervention without the need for more complex and expensive monitoring systems and both Methods 3 and 4 are likely to satisfy this need.

All coverage estimates presented here are for the same calendar period of the women’s group intervention in Bangladesh and, overall, estimates were remarkably close irrespective of method used. Methods 2, 3 and 4 showed only 4% difference in coverage estimates. The close similarity in coverage estimates between these three methods may be explained by the fairly consistent fertility rates reported in national figures in recent years. As a proxy measure, Method 1 estimates coverage among deliveries rather than pregnant women. This method may be subject to recall and reporting biases in recently-delivered women’s self-reports of participation in women’s yet the similarity of this estimate to the estimates derived from the other methods is notable. None of the estimates presented say anything about the equity of intervention coverage or biases in participation in the intervention, although Method 1 has the potential to do so as the survey method enables the collection of background and socio-economic data.

## Conclusion

In the absence of complete and timely population data, choice of coverage estimation method must be based on the strengths and limitations of available methods, capacity and resources for measurement and the ultimate end user needs. Each of the methods presented and discussed here is likely to provide a useful understanding of intervention coverage at a single point in time and Methods 1 and 2 may also provide more reliable estimates of coverage trends. No single method is likely to satisfy all user needs and capabilities and the use of multiple data sources and methodologies are likely to facilitate better understanding of coverage estimates and may stimulate critical review of processes and data where estimates diverge.

## Abbreviations

TBA, Traditional Birth Attendant; M&E, Monitoring and Evaluation; BDHS, Bangladesh Demographic and Health Survey; BBS, Bangladesh Bureau of Statistics; BMMS: Bangladesh Maternal Health Services and Maternal Mortality Survey.

## Competing interests

The authors declare that no competing interests exist.

## Authors’ contributions

All authors contributed to drafting and revising the manuscript. LY and EF prepared the first draft of the manuscript. LY, TH and EF devised methods to estimate coverage. LY and TH conducted data analysis. KA is the project director in Bangladesh and AC is the principal investigator of the evaluation of the women’s group intervention. All authors participated in the interpretation of results and revisions of the manuscript. All authors read and approved the final manuscript.

## Pre-publication history

The pre-publication history for this paper can be accessed here:

http://www.biomedcentral.com/1471-2393/12/60/prepub

## References

[B1] The Millennium Development Goals Report. *United Nations* 2010 [www.developmentgoals.org]

[B2] Lawn JE Cousens S Zupan J 4 million neonatal deaths: When? Where? Why? Lancet 2005 365 9462 891 900 10.1016/S0140-6736(05)71048-5 15752534

[B3] Kerber KJ de Graft-Johnson JE Bhutta ZA Okong P Starrs A Lawn JE Continuum of care for maternal, newborn, and child birth: from slogan to service delivery Lancet 2007 370 1358 1369 10.1016/S0140-6736(07)61578-5 17933651

[B4] WHO, UNICEF & UNFPA Maternal and Neonatal Tetanus Elimination by 2005: Strategies for achieving and maintaining elimination 2000 World Health Organisation Available at: http://www.who.int/vaccines-documents/DocsPDF02/www692.pdf2000

[B5] Victoria C Hanson K Bryce J Vaughan P Achieving universal coverage with health interventions Lancet 2004 364 9444 1541 1548 10.1016/S0140-6736(04)17279-6 15500901

[B6] Bhutta Z Chopra M Axelson H Berman P Boerma T Bryce J Bustreo F Cavagnero E Cometto G Daelmans B Countdown to 2015 decade report (2000–10): taking stock of maternal, newborn, and child survival Lancet 2010 375 9730 2032 2044 10.1016/S0140-6736(10)60678-2 20569843

[B7] WHO World Health Statistics 2010 2010 World Health Organisation Available at: http://www.who.int/whosis/whostat/EN_WHS10_Full.pdf

[B8] Houweling TAJ Azad K Younes L Kuddus A Shaha SK Nahar T Beard J Fottrell E Prost A Costello A The effect of participatory women's groups on birth outcomes in Bangladesh: does coverage matter? A cluster randomised trial Trials 2011 12 208 10.1186/1745-6215-12-208 21943044 PMC3197496

[B9] National Institute of Population Research and Training (NIPORT), ORC Macro, Johns Hopkins University & ICDDR,B Centre for Population Research Bangladesh Maternal Health Services and Maternal Mortality Survey 2001 Dhaka, Bangladesh and Calverton, Maryland USA: NIPORT, ORC Macro, Johns Hopkins University and ICDDR,B

[B10] National Institute of Population Research and Training (NIPORT), Mitra and Associates, Macro International Bangladesh Demographic and Health Survey 2007 2009 Dhaka, Bangladesh and Calverton, Maryland USA: National Institute of Population Research and Training, Mitra and Associates, and Macro International

[B11] Bangladesh Bureau of Statistics Bangladesh Bureau of Statistics 2009 [www.bbs.gov.bd]

